# Anti-mutagenic and Anti-oxidant Potencies of *Cetraria Aculeata (Schreb.) Fr., Cladonia Chlorophaea (Flörke ex Sommerf.) Spreng. *and* Cetrelia olivetorum (Nyl.) W.L. Culb. *&* C.F. Culb.)*

**Published:** 2018

**Authors:** Selcuk Ceker, Furkan Orhan, Selma Sezen, Medine Gulluce, Hakan Ozkan, Ali Aslan, Güleray Agar

**Affiliations:** a *Agri Ibrahim Cecen University, Faculity of Pharmacy, Agri, TR-04100, Turkey. *; b *Agri Ibrahim Cecen University, Central Research and Application Laboratory, Agri, TR-04100, Turkey.*; c *Ataturk University, Faculty of Science, Department of Biology, Erzurum, TR-25240, Turkey. *; d *Yüzüncü Yıl University, Faculity of Pharmacy TR-65150, Turkey.*

**Keywords:** Lichen extracts, Anti-mutagenicity, Anti-oxidant, Short term tests (Ames-*Salmonella*, *E. coli*-WP2, SCE)

## Abstract

In this study, the mutagenic and anti-mutagenic effects of methanol extract of three lichen species (*Cetraria aculeata*, *Cladonia chlorophaea *and *Cetrelia olivetorum*) were investigated by using *E. coli-*WP2, Ames-Salmonella (TA1535 and TA1537) and sister chromatid exchange (SCE) test systems. The results obtained from bacterial test systems demonstrated that methanol extracts of three lichen species have strong anti-mutagenic potencies on TA1535, TA1537 strains and to a lesser extent on *E. coli*-WP2 strain. The anti-oxidant level of human lymphocytes cells was determined in order to clarify the mechanism underlying the anti-mutagenic effects of these lichen species. Co-treatments of 5, 10 and 20 µg/mL concentrations of these three lichen species with AFB decreased the frequencies of SCE and the level of MDA and increased the amount of SOD, GSH and GPx which decreased by aflatoxin. The findings of this work have clearly demonstrated that *Cetraria aculeata*, *Cladonia chlorophaea* and *Cetrelia olivetorum* have significant anti-mutagenic effects which are thought to be partly due to the anti-oxidant activities and the interaction capability of lichen extracts with mutagen agents (Sodium azide, acridin, N-methyl-N′-nitro-N-nitrosoguanidine and aflatoxin B_1_).

## Introduction

Lichens are slow-growing organisms and are a rich source of various biologically active substances used for many purposes. The use of lichen as food, dyes, perfume, species, fodder, and medicine are among these purposes (1, 2). Biological activities of lichens arise from their secondary metabolites. While secondary metabolites of lichen protect/ sustain the symbiotic association from various stress factors (biotic and abiotic), they also have medical applications (3). Anti-microbial (4), anti-inflammatory (5), analgesic (6), anti-viral (7), anti-proliferative (8), anti-oxidant (9-11), and cytotoxic effects (12) are among the applications of lichen secondary metabolites.

In this study, we have investigated anti-mutagenic/anti-genotoxic potential of three lichen species, [*Cetraria aculeata* (Schreb.) Fr., *Cladonia chlorophaea* (Flörke ex Sommerf.) Spreng. and *Cetrelia olivetorum* (Nyl.) W.L. Culb. & C.F. Culb.)]. According to the available literature, *Cetraria* *aculeata* (CA) contains protolichesterinic and lichesterinic acid (13-15), *Cladonia chlorophaea *(CC) contains fumarprotocetraric, homosekikaic, imbricaric and protolichesterinic acid (16) and *Cetrelia olivetorum*  (CO) contains atranorin, chloroatranorin, and olivetoric acid as major contents (16)*. *To the best of our knowledge there is no study about CC and CO lichen species. On the other hand, there are several studies reporting a wide range activity of secondary metabolites of these lichen species. In this respect, Celenza *et al.* (17) reported anti-bacterial activity of fumarprotocetraric acid form a Chilean lichen species. Similarly, Tigre *et al.* (18) reported allelopathic and bio-herbicidal potential of fumarprotocetraric acid. Luo *et al.* (19) indicated that homosekikaic containing an edible lichen species has anti-oxidant and free radical scavenging activities. In a more recent study, anti-microbial and anti-oxidant properties of homosekikaic have been demonstrated (20). Oettl *et al.* (21) isolate two depsides (imbricaric and perlatolic acid) from a lichen species (*Cetrelia monachorum*) and demonstrated that these depsides have multi-targeting anti-inflammatory effects. The studies of lichen secondary metabolites have been performed either with content isolated from lichens or with commercial forms. For example, Backorova *et al.* (22) have purchased four lichen secondary metabolites (atranorin, usnic acid, parietin and gyrophoric acid) and demonstrated that usnic acid and atranorin were more effective than other compounds investigated. In a study performed with three different cancer cell lines, Kristmundsdottir *et al.* (23) reported that (+) -usnic acid has positive effects on all cell lines tested. Valencia-Islas *et al*. (24) reported that boninic acid, 2-O-methylsekikaic acid, salazinic acid, chloroatranorin, atranorin, and (+) -usnic acid have anti-oxidant activity and anti-radical potential. In another study performed with protolichesterinic acid it has been demonstrated that this compound reduce the growth of two different prostate cancer lines (25). 

Taking the knowledge of the literature and the results of this study into account, it seems that the investigated lichen species have significant anti-oxidant and anti-mutagen properties. In light of the fact that the investigated lichen species did not show any genotoxic properties on the Ames-*Salmonella* and human lymphocytes cells, they can be considered as genotoxically safe at all tested concentrations and can be used as promising agents in order to ameliorated toxicity of sodium azide, acridin, N-methyl-N′-nitro-N-nitrosoguanidine, and aflatoxin B_1_.

## Experimental

*Plant material*

Lichen samples of CA, CC and CO were collected from Giresun province of Turkey in 2010. The samples were identified by Associate Prof. Dr. Ali ASLAN, using various flora books and papers (26-28). Vouchers were stored in the herbarium of Kazım Karabekir Education Faculty, Ataturk University (the herbarium number of CA: ATA-KKEF-1865, CC: ATA-KKEF-1866 and CO: ATA-KKEF-1876).

**Table 1:** Anti-mutagenicity assay results of the methanol extract of CA, CC and CO for *S. tyhimurium *TA1535, TA1537 and *E. coli *WP2*uvrA *bacterial tester strains

**Test Items**	**Concentration (µg/plate)**	**Number of revertants**					
		***E. coli *** **WP2** ***uvrA***		***S. typhimurium *** **TA1535**		***S. typhimurium *** **TA1537**	
		**Mean S.E.**	**Inhib. %**	**Mean S.E.**	**Inhib. %**	**Mean S.E.**	**Inhib. %**
Mutagen agent (positive control)	MNNG (1), NaN_3_(1) 9-AA(40)	285.500 07.15		548.00 12.26ö		311.00 07.16ö	
DMSO^**^ (µL/plate)	100	28.00 01.23		12.50 01.00		11.00 01.20	
CA	2	258.50 04.50	**-**	361.50 08.45	34.03	292.00 03.40	**-**
	4	259.50 03.78	**-**	376.50 07.29	31.38	229.00 05.27	26.36
	6	241.00 04.08	**-**	387.50 10.13	29.28	220.00 04.50	29.26
	8	219.50 05.60	**-**	408.50 08.10	25.45	214.00 05.63	31.18
	10	261.00 02.23	**-**	431.00 06.82	21.35	184.00 04.30	40.83
Mutagen agent (positive control)		561.00 01.47		548.00 01.36		347.00 00.85	
DMSO^**^ (µL/plate)	100	23.00 02.04		12.50 01.14		11.00 01.26	
CC	2	350.00 02.70	37.61	371.00 08.40	32.29	251.00 05.48	27.66
	4	362.50 05.00	35.38	397.50 05.45	27.46	243.50 04.27	29.82
	6	396.50 04.10	29.32	331.00 10.00	39.59	203.50 05.16	41.35
	8	334.00 03.06	40.46	408.50 04.22	25.45	198.00 03.34	42.93
	10	372.00 03.88	33.68	441.00 06.45	-	203.50 05.30	41.35
Mutagen agent (positive control)		458.00 04.00		487.00 01.75		347.00 00.74	
DMSO^**^ (µL/plate)	100	33.00 03.17		12.50 01.06		11.00 01.23	
CO	2	355.50 02.50	22.37	319.00 07.18	34.49	212.00 04.40	38.91
	4	368.00 02.18	**-**	302.50 10.00	37.88	255.00 02.51	26.51
	6	389.50 05.42	**-**	341.50 08.20	29.87	253.00 02.57	27.08
	8	386.50 06.00	**-**	389.00 04.50	20.12	188.00 01.90	45.82
	10	376.50 02.60	**-**	353.50 06.27	27.41	163.50 04.35	52.88

* *P *<0,05,

** MNNG, NaN_3_ and 9-AA were used as positive controls for *E. coli *WP2*uvrA*, S*. typhimurium *TA1535 and TA1537 strains, respectively. 10% DMSO (dimethylsulfoxide) was used as negative control. CA: *Cetraria* *aculeata, *CC:*Cladonia chlorophae *and CO: *Cetralia olivetorum. S.E:Standard error.*

**Table 2:** SCE frequency in human blood lymphocytes treated with AFB and CA, CC and CO.

	**Concentrations **	**Metaphase **	**Range of SCEs **	**SCEs/Cell **	**SCE/Cell ± S.E**
**Test Items**					
**Control**		80	2-8	421	6.91 ± 0.82^a^
**AFB**	5 µM	80	6-14	476	9.60 ± 0.21^e^
**CA**	10 µg/mL	80	3-9	406	6.96 ± 0.13^a^
**AFB + CA**	5 µM + 5 µg/mL	80	5-9	508	8.40 ± 0.10^cd^
**AFB +CA**	5 µM + 10 µg/mL	80	3-10	471	7.93 ± 0.32^bc^
**AFB + CA**	5 µM + 20 µg/mL	80	4-10	446	7.43 ± 0.17^b^
**Control**		80	4-7	382	6.36 ± 0.78^a^
**AFB**	5 µM	80	3-13	546	9.10 ± 0.14^e^
**CC**	10 µg/mL	80	4-9	412	6.86 ± 0.12^ab^
**AFB + CC**	5 µM + 5 µg/mL	80	3-11	491	8.18 ± 0.21^d^
**AFB + CC**	5 µM + 10 µg/mL	80	4-10	460	7.63 ± 0.19^c^
**AFB + CC**	5 µM + 20 µg/mL	80	5-10	448	7.46 ± 0.22^bc^
**Control**		80	2-7	332	5.53 ± 0.67^a^
**AFB**	5 µM	80	4-12	536	8.93 ± 0.41^e^
**CO**	10 µg/mL	80	3-9	346	6.10 ± 0.09^a^
**AFB + CO**	5 µM + 5 µg/mL	80	4-10	440	7.41 ± 0.33^cd^
**AFB + CO**	5 µM + 10 µg/mL	80	3-11	400	6.66 ± 0.27^b^
**AFB + CO**	5 µM + 20 µg/mL	80	4-9	394	6.63 ± 0.16^b^

Aflatoxin B_1_ (AFB) was used as positive controls for human blood cells. Dimethyl sulfoxide (DMSO) was used as negative control. Values of each case SCE not sharing a common letter superscript (a, b, c, d, e) are significantly different compared to negative control of each group (*P* <0.05). CA: *Cetraria* *aculeata, *CC:*Cladonia chlorophae *and CO: *Cetralia olivetorum. S.E:Standard error.*

**Table 3:** The effects of AFB and MEL on SOD, GP_X_, GSH and MDA enzymes activities

Test Items	**SOD (U/mL)**	**GPx (U/mL)**	**GSH (µmol/L)**	**MDA (nmol/mL)**
Control	1.53 ± 0.22^a^	1.18 ± 0.07^a^	3.36 ± 0.41^a^	1.30 ± 0.17^a^
AFB (5 µM)	0.97 ± 0.21^d^	0.46 ± 0.12^d^	1.34 ± 0.12^d^	4.69 ± 0.10^d^
CA (10 µg/mL)	1.62 ± 0.18^a^	1.42 ± 0.08^a^	3.12 ± 0.13^a^	2.66 ± 0.20^b^
AFB (5 µM) + CA (5 µg/mL)	1.24 ± 0.08^bc^	0.98 ± 0.11^b^	2.67 ± 0.22^bc^	5.44 ± 0. 12^d^
AFB (5 µM) + CA(10 µg/mL)	1.41 ± 0.16^ab^	1.08 ± 0.10^ab^	2.98 ± 0.16^ab^	3.73 ± 0.21^c^
AFB (5 µM) + CA (20 µg/mL)	1.49 ± 0.21^a^	1.57 ± 0.07^a^	3.22 ± 0.18^a^	3.02 ± 0.27^bc^
Control	1.81 ± 0.12^a^	0.89 ± 0.12^a^	3.92 ± 0.20^a^	3.42 ± 0.08^a^
AFB (5 µM)	0.99 ± 0.43^d^	0.51 ± 0.17^d^	1.37 ± 0.12^d^	4.81 ± 0.13^d^
CC (10 µg/mL)	1.86 ± 0.22^a^	1.44 ± 0.15^a^	3.42 ± 0.14^a^	3.73 ± 0.10^b^
AFB (5 µM) + CC (5 µg/mL)	1.74 ± 0.17^a^	0.77 ± 0.1^b^	2.26 ± 0.19^c^	5.53 ± 0.09^d^
AFB (5 µM) + CC (10 µg/mL)	1.72 ± 0.10^a^	0.78 ± 0.21^b^	3.84 ± 0.21^a^	3.97 ± 0.24^bc^
AFB (5 µM) + CC (20 µg/mL)	1.71 ± 0.35^a^	0.96 ± 0.07^a^	3.78 ± 0.17^a^	3.51 ± 0.32^a^
Control	1.65 ± 0.21^a^	1.38 ± 0.08^a^	3.15 ± 0.20^a^	2.41 ± 0.07^a^
AFB (5 µM)	0.96 ± 0.20^d^	0.44 ± 0.18^d^	1.35 ± 0.07^d^	4.72 ± 0.08^d^
CO (10 µg/mL)	1.63 ± 0.18^a^	1.42 ± 0.25^a^	3.18 ± 0.11^a^	2.98 ± 0.22^b^
AFB (5 µM) + CO (5 µg/mL)	1.67 ± 0.09^a^	0.80 ± 0.09^c^	2.64 ± 0.23^b^	2.34 ± 0.21^a^
AFB (5 µM) + CO (10 µg/mL)	1.69 ± 0.36^a^	0.96 ± 0.06^b^	2.91 ± 0.07^ab^	2.24 ± 0.19^a^
AFB (5 µM) + CO (20 µg/mL)	1.68 ± 0.13^a^	1.18 ± 0.11^ab^	3.05 ± 0.15^a^	4.60 ± 0.12^d^

Aflatoxin B_1_ (AFB) was used as positive controls for human blood cells. Dimethyl sulfoxide (DMSO) was used as negative control. Values of each case (SOD, GPx, GSH and MDA) not sharing a common letter superscript (a, b, c, d) are significantly different compared to negative control of each group (*P* <0.05).

CA: *Cetraria* *aculeata, *CC:*Cladonia chlorophae *and CO: *Cetralia olivetorum. *MEL: Methanol Extract of Lichens.

*Preparation of methanol extracts*

Air-dried and powdered lichens (20 g) were extracted with 200 mL of methanol using the Soxhlet extractor (Isopad, Heidelberg, Germany) for 48 h at a temperature not exceeding the boiling point of the solvent (29). The extract was filtered using Whatman fitler paper (no.1) and then concentrated in vacuum at 40 °C using a rotary evaporator (Buchi Labortechnic AG, Flawil, Switzerland) yielding a waxy material. 

*Cytogenetic analysis of sister chromatid exchange (SCE)*

For the cytogenetic analysis of SCE peripheral blood lymphocytes were taken from four nonsmoking healthy donors between the ages of 22 and 25. Lymphocyte cultures were set up by adding 0.5 mL of heparinized whole blood to RPMI-1640 chromosome medium supplemented with 15% heat-inactivated fetal calf serum, 100 IU/mL streptomycin, 100 IU/mL penicillin and 2% L-glutamine. Lymphocytes were stimulated to divide by 2% phytohemagglutinin. Aflatoxin B_1_ (AFB; 5 µM) and methanol extract of each lichen species (CA, CC and CO) at concentrations of 5, 10 and 20 µg/mL were added to the cultures just before incubation. 

For sister chromatid exchange (SCE) demonstration, the cultures were incubated at 37 °C for 72 h and 5-bromo 2-deoxyuridine at 8 µg/mL was added at the initiation of cultures. All cultures were maintained in darkness. Afterward, 3 µg/mL of colcemide was added 90 min before harvesting to arrest the cells at metaphase. The cultures were centrifuged at 1200 rpm for 10 min. The supernatants were used for enzyme analysis. Cells were harvested and treated for 25 min at 37 °C with hypotonic solution (0.075 M KCl) and centrifuged for 10 min at 1200 rpm then fixed in a 1:3 mixture of acetic acid/methanol (vol/vol). The treatment with fixative was repeated three times. Then, the cells were spread on glass slides and air-dried in dark for 1 week. The staining of air dried slides was performed following the modified fluorescence plus 5% Giemsa method for SCE (30). The slides was irradiated with 30 W, 254 nm UV lamp at 15 cm distance in Sorensen buffer for 30 min, then incubated with 19 SSC (standard saline citrate) at 65 °C for 15 min and stained with 5% Giemsa prepared with Sorensen buffer. Thereafter, the slides were air-dried in dark for 1 week. In order to score SCE, 20 satisfactory metaphases were analyzed. For each treatment condition, well-spread second division metaphases containing 42–46 chromosomes in each cell were scored, and the values obtained were calculated as SCEs per cell.

*Bacterial genotoxicity assays*

*Bacterial strains*

*Salmonella typhimurium* TA1535 (ATCC^®^ Number: 29629), *S. typhimurium* TA1537 (ATCC^®^ Number: 29630) strains were provided by The American Type Culture Collection – Bacteria Department of Georgetown University, Washington, USA, and *E. coli* WP2uvrA (ATCC® Number: 49979) strain was provided by LGC standards Middlesex, UK. All strains were stored at -80 ºC. Working cultures were prepared by inoculating nutrient broth with the frozen cultures, followed by an overnight incubation at 37 ºC with gentle agitation (31).

*Viability assays and determination of test concentrations*

Toxic levels of the test materials towards *S. typhimurium* TA1535, 1537 and *E. coli* WP2uvrA strains were determined as described in detail elsewhere (32). These tests confirmed that there was normal growth of the background lawn, spontaneous colony numbers within the regular range, and no significant reduction in cell survival. Thus, for the concentrations and conditions reported here, no toxicity or other adverse effects were observed.

*Ames-Salmonella and WP2 test systems*

The bacterial mutagenicity and anti-mutagenicity assays were performed as described before (33). The known mutagens NaN_3_ (in distilled water -1 µg/plate) for *S. typhimurium *TA1535 and 9-AA (in methanol -10 µg/plate) for *S. typhimurium* TA1537 were used as positive controls and 10% DMSO was used as negative control in these studies. In the mutagenicity test performed with TA1535 and TA1537 strains of *S. typhimurium*, 100 µL of the overnight bacterial culture, 50 µL test compounds at different concentrations (20, 40, 60, 80 and 100 µg/ /plate in 10% DMSO) and 500 µL phosphate buffer were added to 2 mL of the top agar containing 0.5 mM histidine/biotin. The mixture was poured onto minimal glucose plates. Histidine-independent revertant colonies and viable cells were scored on plates after incubation at 37 °C for 48 or 72 h. In the anti-mutagenicity test performed with the same strains, 100 µL of the overnight bacterial culture, 50 µL mutagen, 50 µL test compounds at different concentrations (20, 40, 60, 80 and 100 µg/ /plate in 10% DMSO) and 500 µL phosphate buffer were added to 2 mL of the top agar containing 0.5 mM histidine/biotin. The mixture was poured onto minimal glucose plates. Histidine independent revertant colonies and viable cells were scored on plates after incubation at 37 °C for 48 or 72 h. The procedures of mutagenicity and anti-mutagenicity assays, which are mentioned for the Salmonella assay, are all applicable to the *E. coli*-WP2 reverse mutation assay. The only procedural difference is the addition of limited tryptophan (0.05 mM) instead of histidine to the top agar (34). The plate incorporation method was used to assess the results of mutagenicity and anti-mutagenicity assays (33, 34). For the mutagenicity assays, the mutagenic index was calculated for each concentration, which is the average number of revertants per plate divided by the average number of revertants per plate with the negative (solvent) control. A sample was considered as mutagenic when a dose-response relationship and a two fold increase in the number of revertants with at least one concentration were observed (35). For the anti-mutagenicity assays, the inhibition rate (%) of mutagenicity was calculated by using the following equation (M: number of revertants/plate induced by mutagen alone, S0: number of spontaneous revertants, S1: number of revertants/plate induced by the extract plus the mutagen). %Inhibition = 1-[(M-S1) / (M-S0)] ×100 25–40% inhibition was defined as moderate anti-mutagenicity; 40% or more inhibition as strong anti-mutagenicity; and 25% inhibition as no anti-mutagenicity (36).

*Biochemical analysis*

*Superoxide dismutase (SOD) assay*

Cu-Zn-SOD activity of the whole blood cell culture supernatant was evaluated by the method of Sun and others (37). In the assay, 2.45 mL of assay reagent [0.3 mM xanthine, 0.6 mM Na_2_EDTA, 0.15 mM nitroblue tetrazolium (NBT), 0.4 M Na_2_CO_3_, and 1 g/L bovine serum albumin] was combined with equal amount of protein from each experimental group (38) and 50 µL xanthine oxidase was added to initiate the reaction. The reduction of NBT by superoxide anion radicals was determined by measuring the absorbance at 560 nm. Cu, Zn-SOD activity was expressed in units of SOD per mg protein, where 1 U was determined as the amount of enzyme causing half-maximal inhibition of NBT reduction. 

*Glutathion (GSH) assay*

GSH levels in the whole blood cell culture supernatant were assessed according to the method described by Anderson *et al.* (39). In a 3 mL cuvette, 750 µL of 10 mM 5-5’-dithio-bis-2-nitrobenzoic acid (DTNB) solution (100 mM KH_2_PO_4_ plus 5 mM Na_2_EDTA, pH 7.5 and GSH-RD, 625 U/L) was combined with equal amount of protein from each experimental group (40). To each sample 150 µL of 1.47 mM ß-NADPH was added after a 3 min incubation period at room temperature. The mixture was rapidly mixed by inversion and the rate of 5-thio-2-nitrobenzoic acid formation was measured photometrically for 2 min at 412 nm. The reference cuvette contained equal concentrations of DTNB and NADPH but no sample. Values were presented as µmol per gram protein. 

*Glutathione peroxidase (GPx) assay*

GPx activity of the whole blood cell culture supernatant was measured by the method described by Paglia and Valentine (40). In the assay, 100 µL of 8 mM NADPH, 100 µL of 150 mM reduced GSH, 20 µL of glutathione reductase (30 units/mL), 20 µL of 0.12 M sodium azide solution and 2.65 mL of 50 mM potassium phosphate buffer (pH 7.0, 5 mM EDTA) was combined with equal amount of protein from each experimental group (38). The samples were incubated for 30 min at 37 °C. 

The reaction was initiated by the addition of 100 µL of 2 mM H_2_O_2_ solution and the conversion of NADPH to NADP was measured photometrically for 5 min at 340 nm. The enzyme activity was expressed as units per g protein using an extinction coefficient of 6.22 × 10^-6^ for NADPH at 340 nm. 

*Malanoaldehyde (MDA) assay*

MDA levels in the whole blood cell culture supernatant were determined spectrophotometrically according to the method described by Ohkawa *et al.* (41). A mixture of 8.1% sodium dodecyl sulphate, 20% acetic acid and 0.9% thiobarbituric acid was combined with equal amount of protein from each experimental group (38). Distilled water was added to the mixture to make the total volume 4mL. This mixture was incubated at 95 °C for 1 h. After incubation, the samples were left to cool under cold water, 1 mL distilled water and 5 mL n-butanol/pyridine (15:1, v/v) were added to the solution and mixed thoroughly. The samples were centrifuged at 4000 rpm for 10 min. The supernatants were separated and measured at 532 nm. 

The level of MDA was calculated from a standard graph made by using different concentrations (1-10 nmol) of 1, 1, 3, 3-tetramethoxypropane and was expressed as µmol of formed MDA mL of serum.

*Statistical analysis*

Statistical significances of the SCE frequencies were determined using a one-way ANOVA analysis followed by a high range statistical domain using Turkey’s test. A level of probability of *p *<0.05 was taken as indicating statistical significance. Results were expressed as mean ± S.E. All experiments were performed in three replicates and data was compared for reproducibility.

## Results

According to the data obtained from the Ames-*Salmonella* TA1535 strain, any concentrations of three lichen extracts tested have no mutagenic property. On the other hand, the extracts of CA and CO lichen species showed anti-mutagenic activity at all concentrations, and the extract of CC showed anti-mutagenic activity at four concentrations tested. Similarly, these three lichen extracts have significant anti-mutagenic properties on Ames-*Salmonella* TA1537 strain. Depending on the increasing concentrations of lichen extracts, the anti-mutagenicity of lichen extracts was reduced in the TA1535 strain while it was increased in the TA1537 strain (Table 1). Interestingly, the results obtained from *E. coli*-WP2 test system showed that the extract of CA has no anti-mutagenicity and the other two lichen extracts (CC and CO) have anti-mutagenic potential. All results were shown in Table 1.

The other short term test performed in this study was SCE. Three lichen extracts reduced the number of SCEs and that reduction was found to be statistically significant (*P* <0.05). The results of SCEs were shown in Table 2.

The final analysis of these three lichen extracts was performed with human lymphocytes cells (the anti-oxidant level of human lymphocytes) in order to clarify the mechanism underlying the anti-mutagenic and anti-genotoxic potential of these lichen species. For this aim, the activities of SOD, GSH, GPx and MDA anti-oxidants of the control and experimental groups were determined. According to the data obtained from human lymphocytes, a decrease in the activities of SOD, GSH, GPx and an increase in the MDA level were observed with treatment of AFB. On the other hand, the application of AFB with different concentrations of lichen extracts increased the activities of SOD, GSH, GPx and decreased the MDA level. The results of the anti-oxidant level of human lymphocytes were represented in Table 3. The statistical analysis showed a significant difference in SOD, GSH, GPx and MDA between all treated samples with AFB and simultaneous treatment of AFB with lichen extracts (*P* < 0.05).

## Discussion

In this study, the mutagenic and anti-mutagenic potential of three lichen species have been investigated and reported that these lichen species have no mutagenic properties, significant anti-mutagenic potentials yet. The investigated lichen species have been examined against MNNG, NaN_3_, 9-AA and AFB mutation agents in the *E. coli*-WP2, Ames-*Salmonella* (TA1535, TA1537) and in human peripheral blood cells, respectively. 

In the *E coli *WP2 test system, the compound, MNNG, was used as a mutagenic agent. This mutagenic agent shows its mutagenic properties by adding alkyl groups to the O^6^ of Guanine, thus, the formation of O^6^-methylguanine occurs. In our study, methanol extract of CC showed a significant anti-mutagenic potential at all concentrations, the methanol extract of CO showed a slight anti-mutagenic potential only at the lowest concentration and the methanol extract of CA did not show any anti-mutagenic effect in the *E coli *WP2 test system against MNNG mutagen agent. The anti-mutagenic activity of methanol extract of CC and CO can be attributed to the inhibitor activities of these lichen extracts on the formation of O^6^-methylguanine as mutagen agent (MNNG) leads to the formation of O^6^-methylguanine.

In the Ames test system, the compound, NaN_3_, was used as a mutagenic agent for the strain of *S. typhimurium* TA1535 strain. The mutagenicity of this compound is to interpose through the production of an organic metabolite (L-azidoadenine) of azide compounds. The generated organic metabolite, L-azidoadenine, enters into nucleus and then interacts with DNA and originates point mutation in the genome (42). Three lichen species have no mutagenic property on *S. typhimurium* TA1535 strain. 

The other strain of *S. typhimurium* used in this study was TA1537. For this strain, 9-AA was used as a mutagenic agent that is known to be a model frameshift agent (43). In the frameshift mutagenesis mechanism, 9-AA binds to DNA non-covalently by intercalation. Through this way, 9-AA induces frameshift mutations at hot spots where guanine is repeated (44). The results obtained from *S. typhimurium *TA1537 strain showed that these three lichen species have no mutagenic properties but anti-mutagenic properties in formation of frameshift caused by 9-AA. When we evaluate the result of the Ames test system, we can conclude that two lichen species (CA and CO) have anti-mutagenic properties at all concentrations and one lichen species (CC) has anti-mutagenic properties at four concentrations against NaN_3_. Similarly, all concentrations of two lichen species (CC and CO) and four concentrations of CA have anti-mutagenicity against 9-AA. The anti-mutagenic activity of three lichen extracts may be due to their inhibition capabilities by blocking the formation of L-azidoadenine and 9-AA binding to DNA.

The third short-term test system used for the evaluation of three lichen species was the formation of SCE in human lymphocyte cells. For SCE test system AFB was used as a positive mutagen agent. AFB is one of the most potent carcinogenic/hepatogenic agents. This compound attacks cellular constitutes such as nucleophilic nitrogen, oxygen, and sulphur heteroatoms (45). When AFB is combined with bases of DNA, it generates adducts which eventually disrupts the process, growth, division, and control of cells (46). Recently, it has been documented that AFB generates micronuclei, SCE, chromosomal aberrations, breakage of chromosomal strands in bone marrow cells and formations of adducts in rodent and human cells (47). The results obtained from human lymphocyte cells demonstrated that all concentrations of CC, CO and two concentrations of CA (20 and 10 µg/mL) reduced the formation of SCE. Interestingly, the lowest concentration of CA increased the formation of SCE. Except from the lowest concentration of CA, other lichen species (CC and CO) and higher concentrations of CA prevented the formation of SCE in a dose dependent manner in human lymphocytes cells which are in accordance with the previous report (47).

On the other hand, a well-known fact is the defense system protection in which cells/organisms are protected against oxidative damage/reactive oxygen species (ROS) by enzymatic and non-enzymatic anti-oxidants. When the anti-oxidant capability of cells is exceeded by the concentration of ROS then oxidative damage endangers the cells or tissue. It is well documented that AFB reduces the level of GSH, activities of SOD and GPx, but it increases the level of MDA (48). In our study the co-treatments of AFB with each of lichen extract (CA, CC and CO) reinstated the level of MDA and the activities of enzymatic and non-enzymatic anti-oxidants (48). Since the anti-oxidants are vital in the preventions of oxidative damage caused by exogenous agents, the use of naturally occurring compounds such as lichens can protect human beings from damage caused by ROS. 

Considering the properties of lichen contents in the literature and the results of this study into account, use of lichen extracts and lichen contents for medical purposes might be useful as they have potential anti-genotoxic properties. On the other hand, more studies are needed; especially clinical studies, which can improve our knowledge about lichen species and their application in related fields.
